# Foot Pad Skin Biopsy in Mouse Models of Hereditary Neuropathy

**DOI:** 10.1002/glia.21069

**Published:** 2010-12

**Authors:** Patrizia Dacci, Giorgia Dina, Federica Cerri, Stefano Carlo Previtali, Ignazio Diego Lopez, Giuseppe Lauria, Maria Laura Feltri, Alessandra Bolino, Giancarlo Comi, Lawrence Wrabetz, Angelo Quattrini

**Affiliations:** Department of Neurology, Division of Neuroscience and INSPE, Neuropathology UnitMilan, Italy; Neuromuscular Diseases Unit, IRCCS Foundation “Carlo Besta” Neurological InstituteMilan, Italy; Division of Genetics and Cell Biology, San Raffaele Scientific InstituteMilan, Italy; Università “Vita Salute”, San Raffaele Scientific InstituteMilan, Italy

**Keywords:** CMT, sural nerve, dermal nerve, epidermal nerve

## Abstract

Numerous transgenic and knockout mouse models of human hereditary neuropathies have become available over the past decade. We describe a simple, reproducible, and safe biopsy of mouse skin for histopathological evaluation of the peripheral nervous system (PNS) in models of hereditary neuropathies. We compared the diagnostic outcome between sciatic nerve and dermal nerves found in skin biopsy (SB) from the hind foot. A total of five animal models of different Charcot-Marie-Tooth neuropathies, and one model of congenital muscular dystrophy associated neuropathy were examined. In wild type mice, dermal nerve fibers were readily identified by immunohistochemistry, light, and electron microscopy and they appeared similar to myelinated fibers in sciatic nerve. In mutant mice, SB manifested myelin abnormalities similar to those observed in sciatic nerves, including hypomyelination, onion bulbs, myelin outfolding, redundant loops, and tomacula. In many strains, however, SB showed additional abnormalities—fiber loss, dense neurofilament packing with lower phosphorylation status, and axonal degeneration—undetected in sciatic nerve, possibly because SB samples distal nerves. SB, a reliable technique to investigate peripheral neuropathies in human beings, is also useful to investigate animal models of hereditary neuropathies. Our data indicate that SB may reveal distal axonal pathology in mouse models and permits sequential follow-up of the neuropathy in an individual mouse, thereby reducing the number of mice necessary to document pathology of the PNS. © 2010 Wiley-Liss, Inc.

## INTRODUCTION

Over the past several years, there has been huge progress in generating transgenic and knockout mice that model aspects of human hereditary neuropathies (Sherer and Wrabetz, 2008). Animal models not only provide formal proof-of-concept for the genetic cause of disease, but they are also important to investigate its progression at the histological level, and its molecular pathogenesis. Transgenic animal models have become essential for exploring novel therapies in Charcot-Marie-Tooth (CMT) neuropathy (Nave et al., 2007).

In addition to the behavioral and functional tests, histological examination of the peripheral nervous system (PNS) is of great importance. Morphological analyses of myelinated nerves have been primarily performed on sciatic nerves. These studies require a surgical approach, and can only be obtained by killing the animals. The study of neuropathy evolution requires time-consuming and expensive analyses of numerous animals. Thus, a more efficient method to evaluate nerve abnormalities by serial analyses of the same animal would be advantageous. Another main limit is that sciatic nerve is a proximal nerve. In inherited peripheral neuropathies, the clinical and pathological alterations are usually more severe in the distal parts of the limbs, especially for features reflecting axonal damage (Barisic et al., 2008; Kamholz et al., 2000; Krajewski et al., 2000; Shy, 2004). Previous reports suggested that sites other than sciatic nerve might be useful for histopathological analysis, such as the nerves of the toes or tail (Bolino etal., 2004; Frei et al., 1999; Wrabetz et al., 2000). Although the analysis of toes is safe and easy, nerves are very thin with small fascicles and only rarely could clear signs of axonal degeneration be detected. Therefore, it is necessary to identify a distal site of nerve examination with the aim to detect alterations similar to those demonstrated on sural nerve biopsy in human beings, including fiber loss and axonal damage.

Although skin biopsy (SB) is rapidly becoming a recognized tool for diagnosing peripheral neuropathy in humans (Holland et al., 1997; Lauria and Lombardi, 2007; Li et al., 2005), its application has been rare in animal models, such as surgical models of nerve injury and models of diabetic or toxic neuropathies (Bianchi et al., 2004; Chiorazzi et al., 2009; Giatti et al., 2009; Navarro et al., 1997; Wu et al., 2001). Thus, we evaluated whether SB might be a satisfactory and reproducible tool to identify nerve abnormalities in mouse models of peripheral neuropathy. We chose a series of animal models with different forms of hereditary neuropathies in which genetic and phenotypic abnormalities have been clearly described (Bolino et al., 2004; Bolis et al., 2005; Nodari et al., 2008; Previtali et al., 2000; Wrabetz et al., 2000, 2006). We performed a SB on the pad (hairless skin) of the hind foot and compared the features of dermal nerves to sciatic nerve. We found that dermal myelinated nerve fibers could be routinely and easily identified in pad SB. Dermal nerves from mutants showed myelin abnormalities qualitatively similar to those seen in sciatic nerve. Moreover, in many cases alterations in dermal nerves were more severe. For example, nerve fibers loss and axonal damage became apparent, even if not detected by sciatic nerve examination. Finally, repeated SB on single animals could allow quantification of progression of disease. Thus, hind foot pad SB in experimental animal models provides the means to recognize pathology earlier and more completely, and to follow the evolution and effects of potential therapies on disease in single animals.

## MATERIALS AND METHODS

### Animals

All of the experiments with animals were performed in the San Raffaele Scientific Institute animal house and are covered by animal protocols approved by the Institutional Animal Care and Use Committee. The animal models analyzed are well established and their PNS phenotypes have been extensively characterized and previously described (Table 1). We investigated different models of P0-related neuropathies derived from altered levels of *Mpz* expression or different *Mpz* point mutations: Congenital Hypomyelination Neuropathy (CHN) (Wrabetz et al., 2000), CMT1B with tomacula (P0-myc, Previtali et al., 2000), CMT1B (S63del) or Déjérine-Sottas Syndrome (DSS, S63C) (Wrabetz et al., 2006); one model of CMT4B (Bolino et al., 2004; Bolis et al., 2005) due to deletion of MTMR2 gene and one model of congenital muscular dystrophy associated neuropathy (MDCN) that consists of a P0Cre/β4 integrin^f/f^-dystroglycan^f/f^, Schwann cell-specific double-null mice (Nodari et al., 2008). Five to ten animals aged 2–12 months were examined per genotype. We compared sciatic nerve to SB samples collected from the ventral aspect (nonhairy glabrous skin or pad) of the hind foot of the mouse.

**Table 1:** Animal Characteristics

CMT neuropathies	Animal model	Phenotype	References
CHN	P0-overexpression	Dysmyelinating neuropathy: Hypomyelination, impaired sorting of axons	Wrabetz et al., 2000
DSS	*Mpz*S63C	Déjérine-Sottas Syndrome: Hypomyelination, few onion bulbs	Wrabetz et al., 2006
CMT1B	*Mpz*S63del	Demyelinating neuropathy: Hypomyelination, florid onion bulbs, demyelination	Wrabetz et al., 2006; Pennuto et al., 2008
CMT1B (tomacula)	P0-Myc	Demyelinating neuropathy: Hypomyelination, uncompaction of myelin, tomacula	Previtali et al., 2000
CMT4B	MTMR2 −/−	Demyelinating neuropathy: Myelin outfolding and recurrent loops	Bolino et al., 2004; Bolis et al., 2005
MDCN	β4integrin-Dg −/−	Demyelinating neuropathy: Abnormal folded myelin: infolding and outfolding, demyelination	Nodari et al., 2008

CMT, Charcot–Marie–Tooth disease; CHN, Congenital Hypomyelination Neuropathy; DSS, Déjérine-Sottas Syndrome; MDCN, Congenital Muscular Dystrophy-associated Neuropathy.

### Skin Biopsy Procedure

**Fig. 1:**
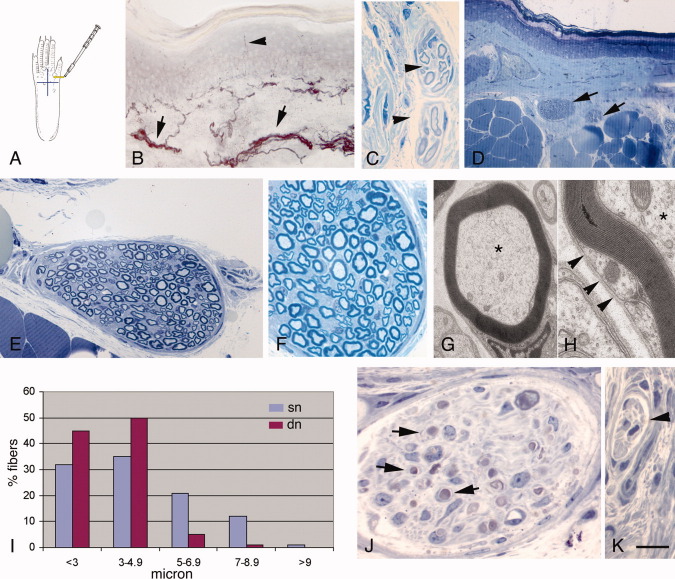
Intraepidermal and dermal nerve fibers in WT mice. A small-sterilized punch was used to taken glabrous skin from the lateral aspect of the palm (**A**). Paraffin sections (**B**) of control skin stained with anti-PGP9 show intraepidermal (arrowhead) and dermal (arrows) nerve fascicles. Transverse semithin sections of small nerve bundles (**C**, arrowhead) run perpendicular to the surface of the skin and large nerve bundle run parallel to the surface of skin (**D**, arrows). Normal myelinated nerve fibers within a large nerve bundle are shown (**E** and **F**). Electron microscopic analysis shows transverse normal nerve fiber (**G**, asterisk marks the axon), normal axonal structure, including neurofilaments and microtubules. At high magnification (**H**) the periodicity of compact myelin appears normal and the myelinated fiber has a tightly apposed basal lamina (arrowheads). Quantification and diameter distribution of myelinated fibers in sciatic and dermal nerves as a percentage of the total number of myelinated fibers (**I**). Transverse semithin sections of dermal nerve after sciatic nerve cut: a large bundle with signs of axonal degeneration (**J**, arrows) and severe fiber loss, a small intraepidermal fascicle with complete loss of fibers (**K**, arrowhead).

Mice were anesthetized with ketamine HCl 200 mg/kg (injected intraperitoneally). Under an operating microscope, the external skin of the foot was sterilized with ethanol. A small sterilized skin punch, 1 mm in diameter and with tip length 8 mm, was introduced perpendicularly to the surface of pad skin, rotated and advanced until reaching 3–4 mm depth (Fig. 1A). The wound was small and was closed with a suture. We encountered no complications and all mice survived the procedure. We observed a fast recovery without any signs of distress. In particular, there was no self-mutilation behavior in the weeks following the procedure.

The size of the biopsied tissue was 1 mm in diameter, which was adequate for histological evaluation of dermal nerve. Only a single foot was biopsied in each procedure and only one SB was done in each mouse. Subsequently, the sciatic nerve from the same animal was removed following euthanasia. To validate the method, however, we performed multiple biopsies (two times) in the same WT animals at different ages (6 and 8 months).

### Morphological Analysis

Histopathological analysis of sciatic and dermal nerves of transgenic and control littermates were performed as described (Lauria et al., 2005; Quattrini et al., 1996). The SB and sciatic nerves of each line of mice was evaluated at age 2–12 months. We performed the following analyses: (i) an immunohistochemical approach, using a rabbit polyclonal antibody (Ab), the pan-axonal protein gene product 9.5 (PGP9.5) (Biogenesis) to identify axons in SB; an immunohistochemical double staining to determine molecular changes of dermal nerves in mutant mice, using the following primary Abs: a panel of neurofilament (NF)-specific Abs, a rabbit polyclonal Ab specific for the poorly or nonphosphorylated NF-H (Chemicon, 1:800), a monoclonal Ab (Abcam, 1:800) specific for highly phosphorylated NF-H and NF-M, a mouse monoclonal Ab (Immunological Science, 1:800) specific for the poorly or nonphosphorylated NF-M; a chicken polyclonal Ab to myelin protein zero (MPZ, Abcam, 1:100); a mouse monoclonal Ab to myelin associated glycoprotein (MAG, Millipore, 1:200) and rabbit polyclonal Ab to contactin-associated protein (Caspr, 1:500) (Peles et al., 1997); (ii) a qualitative morphological analysis to detect the presence of axonal degeneration, axonal regeneration, demyelination, hypomyelination, myelin abnormalities (onion bulbs, myelin outfolding, redundant loops, tomacula); a semiquantitative analysis of histological changes was performed and the amount of pathological alterations was scored: 0 = no signs, + = mild, ++ = moderate, +++ = severe; (iii) quantitative morphometric analysis of semithin sections to quantify the number of myelinated fibers: counting of myelinated nerve fibers was performed in a blind and unbiased manner on an average of three nonoverlapping microscopic fields for CMT1B (P0-myc) and CMT4B mice at different age (CMT1B 6 and 12 months; CMT4B 2 and 6 months; *n* 3 animals for time point). Fibers abnormalities were quantitatively evaluated in mutant sciatic and dermal nerves; (iv) qualitative ultrastructural analysis. Morphometric analysis was performed as previously described (Triolo et al., 2006) and data were analyzed via Student's *t*-test (independent for intertime point analysis and paired for sciatic versus dermal nerve comparisons). The threshold for statistical significance was set at *P* < 0.05.

## RESULTS

### Myelinated Fibers in WT Dermal Nerve

The main phenotypic features of the mouse models used in this study are reported in Table 1. We first validated the SB technique in WT mice. Skin innervation was identified by immunohistochemistry for axons with anti-PGP9.5 antibody. Both superficial unmyelinated intraepidermal nerve fibers and also some deep fascicles were detected in all samples (Fig. 1B). These nerves and fascicles contained not only unmyelinated, but also larger myelinated fibers. The large nerve bundles were usually located in the deep portion of the dermis and traveled parallel to the surface of the skin. The number of bundles varied from one biopsy to another; however, the number and size of bundles was similar among biopsies taken from the same site, as compared to another. Semithin section analysis confirmed the presence of small superficial dermal bundles with few myelinated fibers, running perpendicular to the skin, and large dermal bundles (Fig. 1C,D), containing many myelinated fibers, located between the dermis and sub cutis close to large vessels and adipocytes. Ultrastructural analysis of these nerve fibers showed normal axons and myelin sheaths (Fig. 1G,H).

Morphometric analysis of dermal nerves showed a distribution of nerve fibers similar to sciatic nerve. The dermal nerve fiber density was 9,259 ± 900 per mm^2^ with a G-ratio of 6.6 ± 0.2, with prevalence of small fibers of diameter less than 5 μm (45%) (Fig. 1I). It is known that nerve fibers innervating the plantar pad of the hind paw derive from sciatic nerve. We confirmed these data as sciatic nerve transection was followed by degeneration of nerve fibers in pad SB from the ipsilateral limb (Fig. 1J,K). These data suggest that dermal nerves are easily sampled and possess morphological features similar to those of sciatic nerve.

### Myelin and Axonal Damage in Dermal Nerves from Animal Models of Hereditary Neuropathies

To compare changes in dermal nerves from pad SB to sciatic nerves, we evaluated mutant mice at different ages (Table 2). We detected dermal nerve abnormalities in all mouse models, which resembled the alterations in sciatic nerves. However, pad SB revealed additional abnormal features, such as axonal damage, not previously detected by sciatic nerve examination (Table 2). These mice are presented in detail below.

**Table 2:** Comparison of Sciatic Nerve and Pad Skin Biopsies

	Sciatic nerve											Dermal nerve									
		
	M	Hy	De	OB	UM	To	MO	RL	AFM	AxD	NfA	Hy	De	OB	UM	To	MO	RL	AFM	AxD	NfA
CHN Tg80.4	12	+	0	0	0	0	0	0	0	0	0	+++	0	0	0	0	0	0	0	+	++
CMT1B tomacular Tg88.4	12	+	0	0	+	++	+	0	0	0	0	+	0	0	++	+++	+	0	0	++	0
CMT1B demyelinating Tg129.4	6	+	+	+	0	0	0	0	0	0	+	+++	+++	+++	0	0	0	0	0	++	+
CMT1B hypomyelinated Tg130.3	6	++	0	+	0	0	0	0	0	0	0	+++	+	++	0	0	0	0	0	++	+
CMT4B *Mtmr2*-null	6	0	0	0	0	0	++	++	0	0	0	0	+	+	0	0	+++	+++	0	+	0
MDCN *B4Int/DG*-null	12	0	0	0	0	0	0	0	++	0	0	0	+	+++	0	0	0	0	+++	++	+++

M, months; Hy, hypomyelination; De, demyelination; OB, onion bulb formation; UM, uncompact myelin; To, tomacula; MO, myelin outfoldings; RL, recurrent loops; AFM, abnormally folded myelin; AxD, axonal degeneration; NfA, neurofilament abnormalities. 0 = no signs; + = mild; ++ = moderate; +++ = severe.

**Fig. 2:**
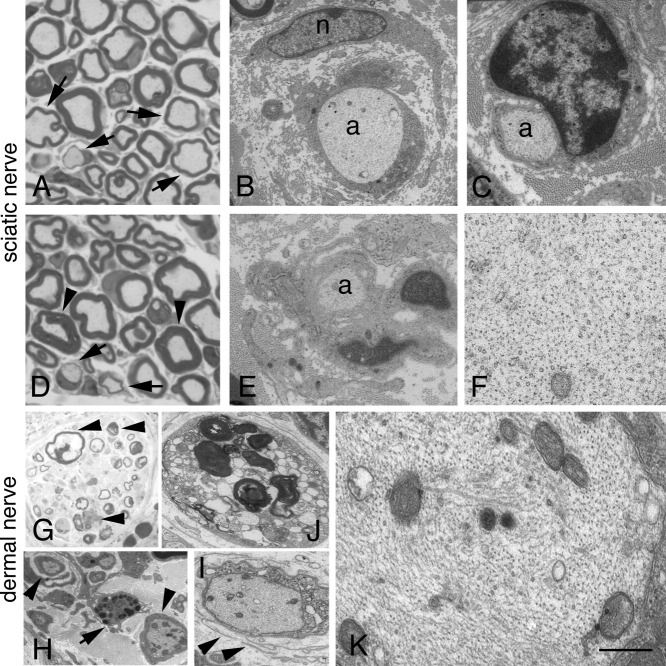
CHN mice develop a severe dysmyelinating neuropathy with axonal degeneration. Sciatic nerve (**A–F**): transverse semithin sections showed some hypomyelinated fibers with some naked large caliber axons (A and B, arrows); some fibers presenting hypermyelination were observed (B, arrowheads). Ultrastructural analysis showed Schwann cell surrounding naked large caliber axons surrounded by redundant basal lamina (C–E). At high magnification a normal organization of neurofilaments in the axon was observed (F). n= Schwann cell nucleous; a = axon. Transverse semithin section of the dermal nerve shows loss of myelinated nerve fibers and signs of acute axonal degeneration (**G**, arrows). Ultrastructural analysis confirmed the presence of axonal degeneration (**J**) with a macrophage inside the basal lamina and shows dysmyelination (**H** and **I**); Schwann cell surrounded naked axon, surrounded by redundant basal lamina (I, arrowheads). At high magnification disorganization of the axon was observed (**K**): transverse section of a dermal axon of a 12-month-old mouse, showing massive accumulation of filamentous aggregates and loss of microtubules.

Mice overexpressing P0 protein developed a severe neuropathy similar to CHN in humans (Wrabetz et al., 2000). P0 overexpression parallels severity of nerve phenotype, among the low (Tg80.3), medium (Tg80.4), and high copy number (Tg80.2). Thus, the 80.2 line manifests severe dysmyelination, hypomyelination and bundles of unsorted, larger diameter axons (data not shown) (Wrabetz et al., 2000). Abnormalities were also present in Tg80.4, and less so in Tg80.3 animals. In this study, to explore whether distal nerve abnormalities are more pronounced, we analyzed the moderately affected Tg80.4. Tg80.4 sciatic nerves were hypomyelinated and hyper-myelinated. Ultrastructural analysis showed dysmyelination (Fig. 2A–E). No abnormalities in axons or axonal degeneration were observed at 6 and 12 months. In contrast, in SB dermal nerves at 6 months, we observed an important reduction of nerve fibers associated with axonal degeneration, not previously detected on sciatic nerve (Fig. 2G–J). Dysmyelinated axons in these mutants have more densely packed NFs, compared to axons in the sciatic nerve (Fig. 2K). Thus, these are typical features of progressive, length-dependent CHN neuropathy.

**Fig. 3:**
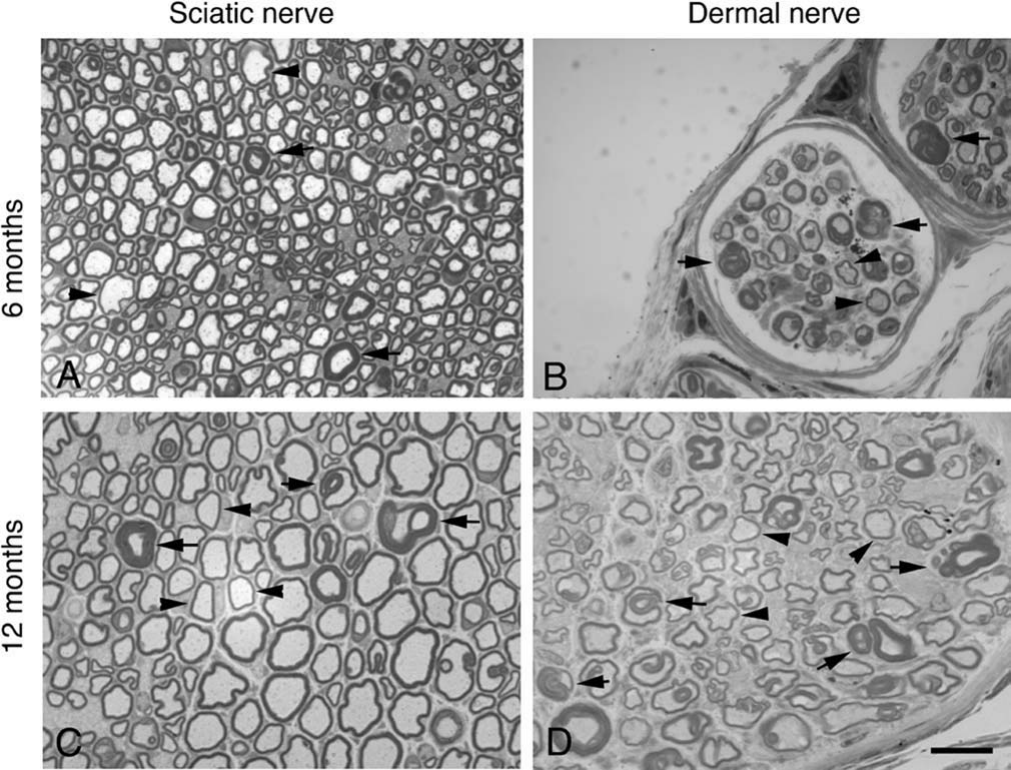
Transverse semithin section of P0-myc mice (CMT1B): in sciatic nerve (**A** and **C**) few tomacula formation (arrows) and hypomyelinated large caliber axons are present (A and C, arrowhead); many tomacula (**B** and **D** arrows) and numerous thinly myelinated (arrowhead) fibers are present in dermal nerves, increasing with age.

P0-myc mice resemble a subtype of CMT1B with tomacula formation (Previtali et al., 2000). P0myc/Tg88.4 mice have mild P0 overexpression. Nonetheless, transverse semithin sections revealed not only diffuse hypomyelination, but also abnormally outfolded myelin sheaths at 6 months (tomacula-like, 2% of fibers), increasing in number with age (Fig. 3). Loss of fibers and signs of axonal degeneration were not present at 12 months in sciatic nerve. In pad SB, we observed similar nerve abnormalities although myelin disruption and tomacula were more numerous. Morphometrical analysis showed lower fiber density and a higher percentage of tomacula-like fibers in dermal nerves (Table 3), reaching a high level of statistical significance at 9 months.

**Table 3:** Quantitative Data: Comparison of Sciatic Nerve and Pad Skin Biopsies

		Fibers # mm^2^, mean (SD)			% of nerve fibers abnormalities, mean (SD)		
					
	M	Sciatic nerve	Dermal nerve	% reduction	Sciatic nerve	Dermal nerve	% difference
CMT1B tomacular: P0-myc	6	8919 (1110)	7961 (1438)	9.59^ns^	1.90 (0.20)	6.87 (1.45)	4.97*
	9	7986 (179)	5335 (193)	26.51***	3.30 (0.53)	8.33 (0.51)	5.03***
CMT4B *Mtmr2*-null	2	9372 (417)	8438 (340)	9.34***	6.00 (5.80)	11.23 (0.75)	5.23**
	6	8742 (646)	7236 (668)	15.06***	16.47 (1.36)	33.60 (2.90)	17.13*

M, months; ns, not significant.

*P* < 0.05

*P* < 0.005

*P* < 0.0005.

**Fig. 4:**
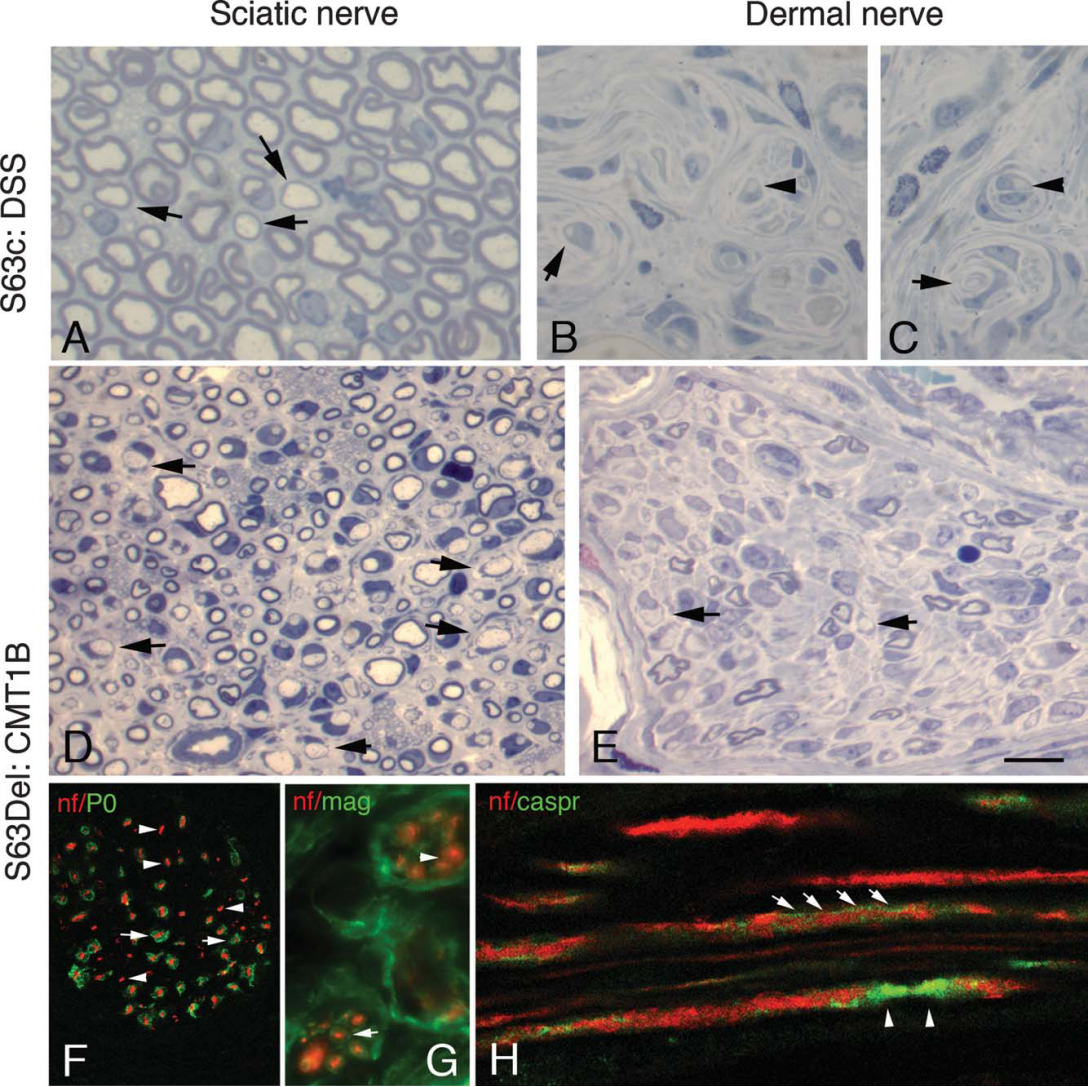
DSS (S63C, 6-months-old). Semithin section of sciatic nerve showed many fibers with thin myelin (hypomyelination) (**A**, arrows); more evident in dermal nerves: hypomyelination with naked axons (**B** and **C**, arrowhead) and onion bulb formations (B and C, arrows). CMT1B (S63del). Semithin section of sciatic nerve (**D**) showed severe hypomyelination with many large caliber axons without myelin (D, arrows); semithin section of dermal nerve (**E**) showed a severe hypomyelination (arrows) and fiber loss. Immunohistochemistry on transverse dermal nerves from 6-months-old S63Del mice using anti-NF (**F**–**H**, red, to recognize axons), anti-P0 antibody (F, green, to recognize compact myelin); anti-MAG antibody (G, green) to detect noncompact myelin and anti-Caspr (H, green), which identifies paranodal domains in longitudinal section: most fibers are positive for P0 (F, arrows) but some large fibers had no detectable P0 staining (arrowhead) in S63del mice; in MAG-stained transverse sections, few fibers were stained around their entire circumference (G, arrows), but others were negative for MAG (arrowhead); in H, Caspr dislocated into paranodes (arrowhead) and internodal region (arrows).

We previously reported that both P0S63del and P0S63C mice develop evident demyelinating neuropathy, with characteristics respectively of CMT1B and DSS (Wrabetz et al., 2006). Lines with higher expression develop more obvious defects. Semithin section analysis of sciatic nerve from 12-month-old S63del and S63C mice show dysmyelinating neuropathy, consisting of hypomyelination and demyelination with onion bulb formation (greater in S63del), without signs of axonal damage. To examine whether a more pronounced phenotype would be detected in the distal part of PNS, we chose line 129.4 and line 130.3, each with milder sciatic nerve phenotypes. Analysis of 6-month-old S63del Tg129.4 mice, revealed hypomyelination and more onion bulbs than S63C Tg130.3, which manifested hypomyelination and less onion bulbs in sciatic nerve (see Fig. 4). In pad SB, in both lines, we confirmed dysmyelinating findings, but features were more severe and frequent than in sciatic nerve, including demyelination and onion bulbs in both lines. We also noted reduced numbers of myelinated fibers and occasional axonal degeneration (see Fig. 4). The axonal damage might result from the proximal process of dysmyelination.

**Fig. 5:**
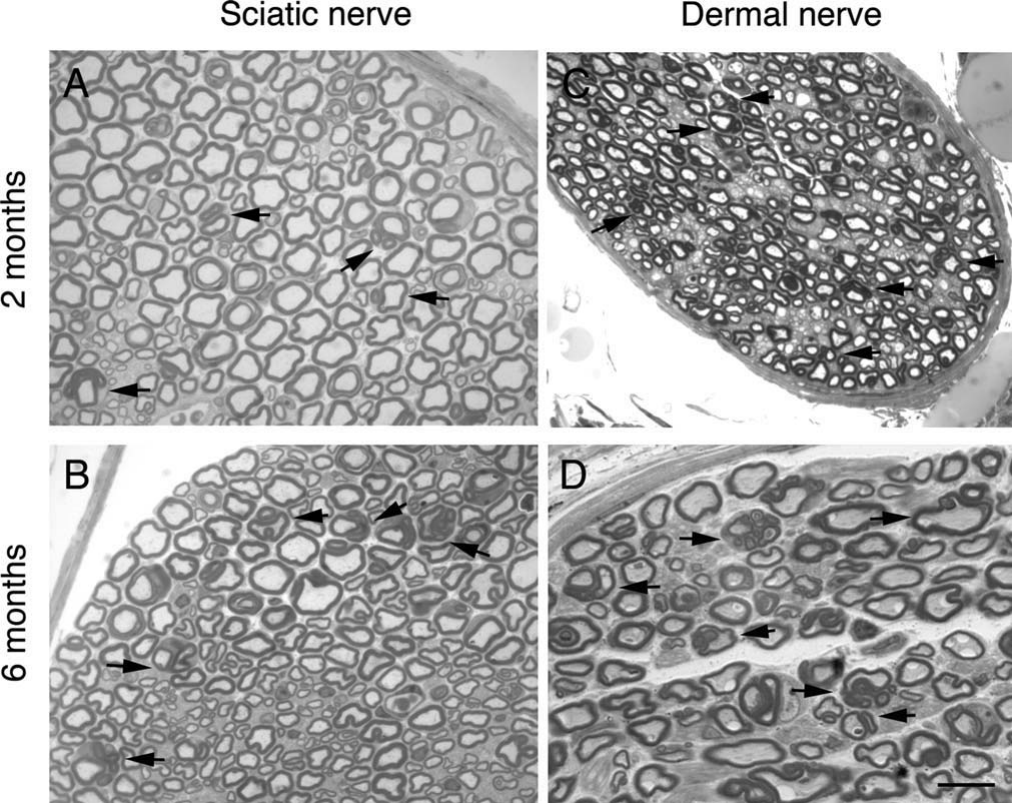
Excessive myelin outfolding in dermal nerves of Mtmr2-null mice. Transverse semithin sections of Mtmr2-null sciatic nerves (**A** and **B**) and dermal nerves (**C** and **D**). Arrows indicate fibers with myelin outfoldings. Myelin outfoldings are more evident and numerous in dermal nerves (C and D), increasing with age.

Schwann cell *Mtmr2*-null mice show myelin outfoldings and redundant loops of myelin increasing with age (5% of myelinated fibres at 2 months, 10% at 4 months, 16% at 6 months, and 26% at 12 months) (Bolino et al., 2004; Bolis et al., 2005). The complexity of the dysmyelinating neuropathy also increased with age as revealed by counting the number of fibers showing three or more satellite loops. In this study, semithin section analysis confirmed the dysmyelinating neuropathy at 2 and 6 months (see Fig. 5). The analysis of nerves from pad SB showed a more severe neuropathy with more numerous signs of dysmyelination and myelin outfoldings (see Fig. 5). In addition, we noted reduced numbers of myelinated fibers, occasional degenerating axons and more evident onion bulbs at 6 months. Dermal nerve fiber density was significantly lower as compared to sciatic nerve at both 2 and 6 months of age (Table 3). The number of fibers containing myelin outfoldings and loops was significantly higher in dermal nerves of *Mtmr2*-null mice examined at 2 and 6 months (Table 3), with more complex myelin outfoldings, as compared to sciatic nerves. These data indicate that the morphological alterations are length dependent.

**Fig. 6:**
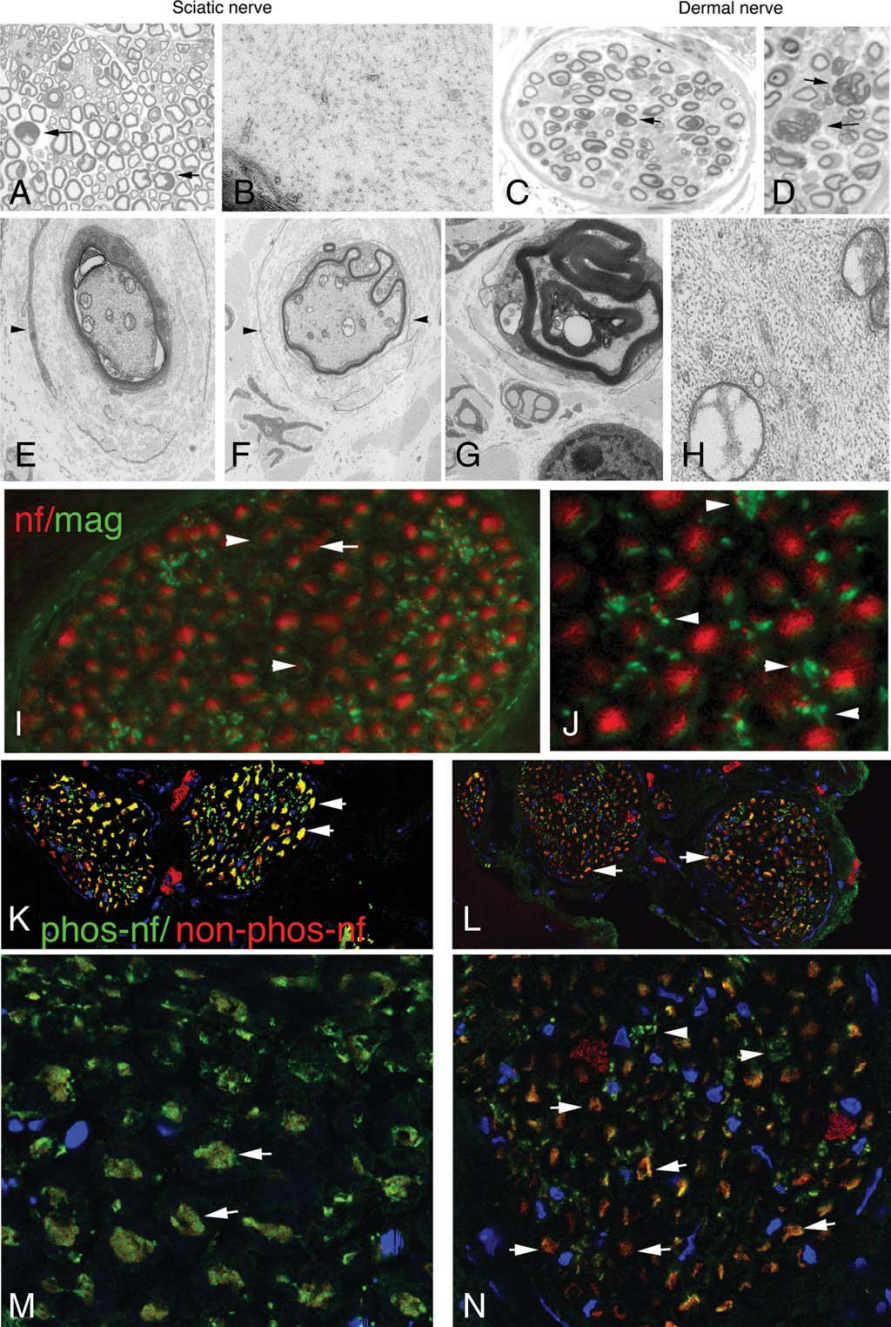
Transverse semithin section of β4integrin/dystroglycan mutants (MCDN) mice: few fibers with myelin abnormalities are present in sciatic nerve (**A**, arrows); abnormalities are severe in dermal nerve (**C** and **D**): numerous abnormal loops, infoldings and outfoldings (arrows) are present. Electron microscopy analysis of dermal nerves of a 12-month-old mouse confirmed chronic remyelination (onion bulbs, **E**–**G**). Electron microscopy showed neurofilamentous accumulations and enlarged mitochondria with varying degrees of loss of structural integrity (**H**) compared to sciatic nerve (**B**). Immunocytochemical localization of MAG in cross sections from MCDN mice (**I** and **J**); MAG staining (green) is variable in intensity and localization and often absent around axons (red) (I, arrow) and in others less intense (I, arrowhed), in J, periaxonal spots of staining (arrowhead), corresponding to myelin loops and outfoldings. Double immunohistochemistry on transverse dermal nerves stained for poorly or nonphosphorylated NF-H (red) and phosphorylated NF-H (green) in WT (**K** and **M**) and MCDN (I and **N**) dermal nerves: double staining shows intense reaction for phosphorylated NF-H in WT mice (K and M, arrows), whereas high positivity for nonphosphoryated NF-H in MCDN dermal nerves (I and N, arrows). Note the green staining for unmyelinated axons (N, arrowhead). Images were acquired using the same settings in WT and MCDN dermal nerves.

α6β4 integrin and dystroglycan cooperate to stabilize the myelin sheaths (Moore et al., 2002). Nodari et al. (2008) showed that sciatic nerves from β4 integrin/dystroglycan double mutants present abnormally folded myelin, with abnormal loops, infolding, and outfolding, which increase in number with age. We confirmed these findings in sciatic nerve. SB, however, revealed a more severe neuropathy with abnormal loops, infolding, and outfolding and associated demyelination and florid formation of onion bulbs, indicating chronic cycles of demyelination and remyelination. In distal axons at 12 months, NFs were closely packed and unevenly distributed, in contrast with the regularly spaced, parallel arrays of NFs in axons in the sciatic nerve (see Fig. 6). Distal SB axons also revealed evident vesicle accumulation and mitochondria abnormalities. Fiber loss was present (50% loss) associated with axonal degeneration. Thus, this mouse model showed all the features of progressive, length-dependent MDCN associated neuropathy as in human (Di Muzio et al., 2003).

### Molecular Changes in Dermal Nerves

We performed immunohistochemical studies to investigate molecular changes in axons and Schwann cells of dermal nerves from CMT1B and MDCN mice. SB from CMT1B (S63del) mice demonstrate a severe dysmyelinating neuropathy (above). By immunohistochemical analysis, we confirmed a variation in the intensity of P0 staining among different fibers (Fig. 4F) with few fibers showing normal staining. Many P0-negative fibers were observed. In MAG-stained sections, usually, fibers had abnormal reaction or no detectable signal (Fig. 4G). Furthermore, in these mice we documented an abnormal localization of Caspr, a paranodal marker, in longitudinal sections with a diffusion of Caspr into the internodes (Fig. 4H), confirming the importance of a normal myelin for the integrity of the nodal/paranodal region.

We have shown that in the distal aspects of MDCN mice axons are significantly altered with abnormal spacing of NFs. It is known that axonal properties, such as spacing and phosphorylation of NFs are dependent on glial characteristics and as has been described, for example, in MAG-deficient mice (Yin et al., 1998). First, we showed in MAG-stained sections from MCDN mutant nerves an abnormal pattern of expression that was irregular among and within individual fibers. Some large axons were negative for MAG staining and others manifested an irregular staining with many dots corresponding to folded myelin, abnormal loops, and outfolding typically observed in this mutant. Then, we observed by immunohistochemistry a reduction of NF-H phosphorylation status in dermal nerve axons in this mutant, confirming the importance of the link between the Schwann cell phenotype and axonal properties.

## DISCUSSION

SB has been exploited to demonstrate the decreased number of epidermal unmyelinated nerve fibers in patients with diabetes (Kennedy et al., 1996; Polydefkis et al., 2001), HIV infection (Polydefkis et al., 2002), and small fiber sensory neuropathy (Devigili et al., 2008; Holland et al., 1997; Kennedy and Wendelschafer-Crabb, 1993). It has also been used to obtain tissue samples from experimental models of peripheral neuropathies such as diabetic and traumatic neuropathy (Giatti et al., 2009; Navarro et al., 1997; Wu et al., 2001). Here, we studied the histopathological alterations in models of hereditary neuropathies using punch SB from the hind foot pad and we compared it to the traditional sciatic nerve examination.

### Dermal Myelinated Nerves Show Morphological Features Similar to Those of Sciatic Nerve

SB was found to be safe, even for mice with very severe neuropathy, reproducible and fast, and usually this method involves gentle and brief handling; thus exposing the animals to minimal stress. An important benefit is that the specimen includes not only the intraepidermal nerve fibers, but also a whole cross section of the deep dermal nerves with myelinated fibers. SB gives sufficient nerve tissue for histopathological analyses and produces good quality results that allow the monitoring and grading of the neuropathy. We have shown that myelinated nerve fibers can be easily and routinely identified and evaluated in SB: dermal nerves appeared by light and electron microscopy identical to myelinated nerve fibers obtained in sciatic nerve analysis. They represent mixed sensory-motor fibers, as suggested by morphometric analysis where they revealed a composition very similar to that of sciatic nerve. Furthermore, they are distal branches of sciatic nerve, as demonstrated by all fibers undergoing axonal degeneration after transection of sciatic nerve. Taken together, the results demonstrate that the structure and organization of myelinated nerves in SB is similar to those of the sciatic nerves. This opens a new, noninvasive approach to study and follow transgenic or genetically engineered strains of mice that serve as animal models for hereditary neuropathies.

### SB Is a Tool to Assess the Neuropathy Severity and Progression

SB revealed many of the morphological abnormalities described in mouse models of hereditary neuropathies. Evaluation of large deep dermal nerves were able to identify hypomyelination in CHN (Wrabetz et al., 2000), myelin abnormalities such as onion bulbs and tomacula in CMT1B, DSS (Previtali et al., 2000; Wrabetz et al., 2006) and MDCN (Nodari et al., 2008) and myelin outfolding in CMT4B (Bolino et al., 2004; Bolis et al., 2005). Our SB approach provided reproducible results, allowing the degree of neuropathy and severity to be scored by light and electron microscopy studies. Moreover SB was less invasive than sciatic nerve investigation, which requires surgery and the death of the mouse. Instead, we showed that SB can be repeated at least two times, in WT animals, and tissue changes could be reliably followed in one animal. In this way, the number of animals employed and costs can be reduced. In all types of mutant mice examined here, SB revealed similar, yet more severe and relevant signs of the underlying pathology, suggesting that punch SB could be performed in alternative to sciatic nerve examination in animal models of hereditary peripheral neuropathies.

We mostly studied models of demyelinating peripheral neuropathies where fiber loss and signs of axonal degeneration are very difficult to detect in the sciatic nerve. In hereditary neuropathies, a frequent cause of disability is progressive axonal degeneration and fiber loss from a dying back process (Barisic et al., 2008; Kamholz et al., 2000; Krajewski et al., 2000; Shy, 2004). We and others have previously studied different distal sites of peripheral nerve damage, such as the nerves of the toe (Bolino et al., 2004; Frei et al., 1999; Wrabetz et al., 2000). The limit of this procedure, in our experience, was that the nerve is very thin with small fascicles, variable branching along the proximal-distal axis, and higher susceptibility to traumatic injury. Instead, pad SB was more easily able to reveal axonal degeneration, not previously detected by sciatic nerve examination. In our models, punch biopsy revealed an important reduction of fibers, increase numbers of abnormalities, ovoids of degeneration suggesting an active axonal damage in the distal part of the PNS. Quantitative data confirmed the gradient of severity of neuropathology from distal to proximal along the fiber length. This provides further evidence that dysmyelination causes secondary axonal damage in models of hereditary neuropathies (Frei etal., 1999).

The maintenance of the Schwann cell-axon unit is essential for axonal integrity. Mice null for MAG, an adhesion molecule that is localized in noncompact myelin, show an abnormal NF network and axonal degeneration in motor nerves, suggesting that MAG is essential for maintenance of both myelin and axonal integrity (Yin et al., 1998). Here, we observed that distal axons in P0 mutant and MDCN mice have abnormalities such as densely packed NFs. SB provided information on the molecular architecture of dermal nerve fibers, in particular showing a decrease in NFs phosphorylation status of distal axons, further supporting the concept that an impaired Schwann cell phenotype can lead to altered axonal properties in demyelinating neuropathy (Martini, 2001). In addition, we found an increased numbers ofmitochondria and intra-axonal vesicles, suggesting animpairment of anterograde axonal transport. One possibility is that shorter internodes, characteristic of dermal myelinated nerves, could increase apparent axonal abnormalities due simply to increase numbers of nodes of Rancier where mitochondria and intra-axonal vesicles are typically found. However, we doubt this as we found such changes in regions of the axon without morphological features of node/paranode.

A common hallmark of many neurodegenerative diseases (Cleveland, 1999; Julien and Beaulieu, 2000), including the hereditary axonal neuropathies (Bomont et al., 2000; Lupski, 2000; Mersiyanova et al., 2000), is disorganization of the NF network with impairment of axonal transport. Transport of organelles and molecules assembled in the neuronal cell body must occur over significant distances in long axons. Furthermore, NFs accumulation or changes may slow axonal transport and perturb the function of microtubules, which are key organelles of intracellular transport (La Monte et al., 2002; Puls et al., 2003). We found abnormalities prevalently in SB, suggesting an impairment of axonal transport that could probably contributes significantly to the ultimate degeneration of axons.

Our findings are in agreement with the results obtained from SB in patients with CMT hereditary neuropathies (Li et al., 2005; Sabet et al., 2006). In fact, dermal nerves from CMT1A and CMT1X patients not only show abnormalities previously detected in sural nerve biopsies, but also detect other abnormal findings previously unreported in CMT. Similarly, in dermal nerves from SB, we observed axonal pathology undetected in our mouse models of hereditary neuropathies. Our data demonstrate that SB is a valid approach to reveal the amount of axonal loss in the distal part of the PNS. In conclusion, SB might be useful to evaluate pathological abnormalities in many animal models of dysmyelinating neuropathies.
